# Studyholism and Study Engagement: What about the Role of Perfectionism, Worry, Overstudy Climate, and Type of School in Adolescence?

**DOI:** 10.3390/ijerph18030910

**Published:** 2021-01-21

**Authors:** Yura Loscalzo

**Affiliations:** Department of Health Sciences, School of Psychology, University of Florence, 50135 Florence, Italy; yura.loscalzo@gmail.com

**Keywords:** GPA, heavy work investment, OCD, school, study addiction, study engagement, work addiction, workaholism

## Abstract

This study aims to deepen the analysis of Studyholism (or obsession toward study) on a sample of 793 Italian adolescents (*M*age = 16.30 ± 1.73; 53% boys). A path analysis model including potential antecedents (i.e., worry, study-related perfectionism, perfectionistic strivings and concerns, overstudy climate, type of school) and outcomes (e.g., grade point average, time spent studying, psychological well-being) of Studyholism was tested. In line with previous findings on college students, this study supported the conceptualization of problematic overstudying as an OCD-related disorder, since worry is the strongest predictor of Studyholism. Moreover, among the main findings, it confirmed that Studyholism is associated with negative academic outcomes, while Study Engagement predicts positive academic outcomes. Finally, it also confirmed that both Studyholism and Study Engagement predict social impairment. In conclusion, preventive interventions to favor students’ academic success and well-being should reduce perfectionistic concerns and study-related perfectionism and enhance time management skills (in Engaged students too). For reducing Studyholism, instead, the primary target should be trait worry. Finally, preventive interventions should be implemented across all the school types and possibly during childhood or pre-adolescence to avoid the increase in psychological and social impairment that has been found to be associated with Studyholism in youths. It is also essential to detect potential early risk indicators (especially among individual factors) of Studyholism in childhood.

## 1. Introduction

Loscalzo and Giannini [1] and Atroszko, Andreassen, Griffiths, and Pallesen [2] recently introduced in the literature a new potential clinical condition associated with problematic overstudying. They defined it, respectively, as Studyholism and Study Addiction. Besides the difference in the term chosen to define this new potential clinical condition, there are critical theoretical differences between the two constructs, as highlighted in previous papers by Loscalzo and Giannini [1,3,4,5,6,7].

In sum, Atroszko et al. [2] conceptualized problematic overstudying using the behavioral addiction framework; hence, they suggested that it is a behavioral addiction characterized by the seven core components of substance addictions (i.e., salience, tolerance, mood modification, relapse, withdrawal, conflict, and problems). Loscalzo and Giannini [1], instead, went beyond the a priori assumption of addiction and analyzed problematic overstudying in all its potential manifestations, aiming to discover its real (externalizing and/or internalizing) nature, as suggested by Kardefelt-Winther [8]. Hence, they considered the possibility that problematic overstudying could be characterized by both addiction and obsessive symptoms, and either high or low study engagement. Therefore, they suggested that some Studyholics could also have a positive attitude towards their study. In line with this, using the Heavy Study Investment framework to avoid an overpathologization of a common behavior such as studying (see Billieux et al. [9]), they distinguished between three types of Heavy Study Investors, two of which are Studyholics: Disengaged Studyholics, or students with high levels of Studyholism and low levels of Study Engagement; Engaged Studyholics, who have high levels of both Studyholism and Study Engagement; and Engaged students, namely students with low levels of Studyholism and high Study Engagement. Finally, Loscalzo and Giannini [1] suggested a comprehensive model that, based on workaholism literature [10], listed potential antecedents and outcomes, which they distinguished as individual and situational.

Next, based on the results highlighted by the analyses on the Studyholism Inventory (SI-10) [11] and by Loscalzo and Giannini’s [5] study, which tested many of the potential antecedents and outcomes of Studyholism on a wide sample of Italian college students, Loscalzo and Giannini [1,3,4,5,6,7] concluded that Studyholism is more similar to an obsession with study, than to an addiction to study. However, they pointed out that more research was needed to uncover the real nature of problematic overstudying. Finally, recently, Loscalzo and Giannini [7] suggested their Studyholism DSM-like *tentative* criteria, which state that Studyholism is characterized by persistent and recurrent problematic studying behaviors that cause clinically significant impairment/distress and, more specifically, by study-related obsessions and/or study-related compulsions during the last six months. Moreover, besides the exclusion criteria concerning the physiological effects of a substance or a medical condition and other mental disorders, there are also two specifiers: (i) study engagement level (high, average, low); (ii) major area of impairment (academic, social, academic and social).

In conclusion, Loscalzo and Giannini [1] coined the term “Studyholism”, despite the fact that in the literature the term “Study Addiction” was already present, since it does not include the word “addiction”, which could favor a reduction of the construct to addiction and negative components only. In fact, they stressed that Studyholism might be more similar to an obsession and might also be associated with Study Engagement, namely a positive dimension. Moreover, as highlighted in a subsequent paper [3], they aim to avoid that problematic overstudying will be characterized by the confusion surrounding the literature about problematic overworking: many different definitions and theorizations have been used referring, often interchangeably, to workaholism and work addiction. Therefore, Loscalzo and Giannini [3,4] used the term “problematic overstudying” when referring to the problematic behavior generally—without referring to a specific theorization—while they use “Study Addiction” for the behavioral addiction conceptualization and “Studyholism” for the obsessive-compulsive (OCD)-related disorder conceptualization.

Since Studyholism is widespread in both Italian youths [12] and adolescents [13], and taking into account the negative outcomes associated with this new potential clinical condition in youths [5], it is critical to replicate Loscalzo and Giannini’s [5] study on a sample of Italian adolescents. This will allow for detecting the similarities and differences that might exist between youths and adolescents concerning the relationship between Studyholism (and Study Engagement) and its potential antecedents and outcomes, with implications for preventive and clinical interventions.

### 1.1. Loscalzo and Giannini’s Study on Youths

Loscalzo and Giannini [5] gathered a sample of 1958 Italian college students who were heterogeneous concerning the year of study, the major of study, and the city of residence. Next, they performed a path analysis to test many of their suggested antecedents and outcomes [1]. Through this analysis, they found support for their definition of Studyholism as an OCD-related disorder since worry, which is a transdiagnostic process across internalizing disorders, including OCD [14,15,16], proved to be a very strong predictor of Studyholism (*β* = 0.67). Instead, perfectionism does not predict Studyholism in any of the three components they analyzed (i.e., perfectionistic strivings, perfectionistic concerns, study-related perfectionism), in contrast with the workaholism literature [17]. Hence, in line with Loscalzo and Giannini [1,18], they suggested that problematic overworking and problematic overstudying, despite their similarities, could also have some differences. Therefore, they should be analyzed as different constructs without merely applying workaholism/work addiction to the school context. However, Loscalzo and Giannini [5] specified that more studies on the link between Studyholism and perfectionism are needed to deepen this relationship. Regarding Study Engagement, it is positively predicted by perfectionistic strivings and study-related perfectionism, while perfectionistic concerns negatively predict it. Moreover, regarding situational antecedents, they found that school and family overstudying climates do not predict Studyholism and Study Engagement. However, they suggested analyzing the effect of this antecedent on both Studyholism and Study Engagement in pre-adolescents and adolescents. In fact, Loscalzo and Giannini [1] speculated that Studyholism could have its onset from pre-adolescence, due to the increase in the time and effort required by school around this stage, and that the influence of parents’ and teachers’ overstudying climate might be stronger in adolescents than in youths. Finally, the area/major of study predicts neither Studyholism nor Study Engagement.

Regarding academic outcomes, Loscalzo and Giannini [5] found that Studyholism does not predict grade point average (GPA), while Study Engagement is a positive predictor of academic performance. Moreover, both Studyholism and Study Engagement positively predict time spent studying generally and before exams. Finally, dropout intention is predicted by both Studyholism (positively) and Study Engagement (negatively). Concerning psychological outcomes, Studyholism does not predict positive affect, while it positively predicts negative emotions and stress. Study Engagement, instead, is a positive predictor of positive affect and a negative predictor of stress. With regard to physical outcomes, Studyholism is a positive predictor of sleep quality impairment and daytime sleepiness, while Study Engagement reaches the (selected) *β* value cut-off of 0.10 for daytime sleepiness only (being a negative predictor). Finally, for social outcomes, neither Studyholism nor Study Engagement predict aggressive behaviors at university. Loscalzo and Giannini [5] suggested that this (situational) social variable should be analyzed further in younger students. Finally, family and friends’ complaints due to study and social relationship impairment due to study are positively predicted by both Studyholism and Study Engagement, suggesting that both types of Heavy Study Investment are associated with social impairment.

Loscalzo and Giannini [5] also looked for potential differences between Engaged and Disengaged Studyholics in the antecedents and outcomes they analyzed. They found that in Engaged Studyholics, perfectionistic strivings and study-related perfectionism are higher, while perfectionistic concerns are lower than in Disengaged Studyholics. Instead, the two types of Studyholics do not differ concerning worry and overstudying climate (both at school and in the family). Moreover, regarding the outcomes, there is no difference in sleep quality, daytime sleepiness, and stress between the two types of Studyholic. However, Disengaged Studyholics have lower positive affect and higher negative affect than Engaged Studyholics. Concerning social outcomes, the Engaged Studyholic type is more impaired, since these students have higher family and friends’ complaints due to study and higher social relationship impairment due to study than Disengaged Studyholics. There is no difference in quarrels at university between the two types of Studyholic. Finally, regarding study-related outcomes, Disengaged Studyholics have higher dropout intention and lower GPA than Engaged Studyholics. In addition, Engaged Studyholics spend more time studying (both generally and before exams) than Disengaged Studyholics. In sum, Disengaged Studyholics are more impaired in the affective and academic areas, while Engaged Studyholics are more socially impaired.

### 1.2. The Present Study

Loscalzo and Giannini’s [5] study represents the first attempt to shed light on the internalizing/externalizing nature of Studyholism, as well as on its potential antecedents and outcomes. Hence, it is critical to analyze these variables further, and also on a sample of younger students, since there might be some differences in both the antecedents (worry, perfectionism, overstudying climate) and outcomes (academic, physical, psychological, and social).

The analysis of Studyholism in adolescents is imperative, since Erickson [19] stated that puberty is a sensitive stage of life characterized by the direction of energies toward sport, artistic activities, and school. Moreover, this phase of life encompasses a critical challenge in adolescents: they make a transition from secondary school of first grade to secondary school of second grade and, besides encountering new teachers and classmates, they face the reality of the field of study they have chosen in the previous year [20], which might be different from their expectations concerning both the time and the energy to devote to study.

The Italian school system comprises the following school levels until adolescence: day nursery (0–3 years), kindergarten (3–6 years), primary school (6–11 years), secondary school of first grade (11–14 years), and secondary school of second grade (14–19 years) [21]. Regarding secondary school of second grade, students can choose among three types: High School, which is divided into six curricula (artistic, classical, linguistic, music and dance, scientific—also with the “applied sciences” option, human sciences—also with the “socio-economic” option) [22]; Technical Schools, which offer training related to two main areas (i.e., economy and technology), which are divided into 11 curricula and that are fundamental for the country’s economic and productive development [23]; Professional Schools, which provide a large amount of laboratory activities, as they train students in art, crafts, and professions that are strategic for the country’s economy (e.g., industry and craftsmanship for products made in Italy; auxiliary arts of the health professions, such as dental technicians), including 11 curricula [24].

In Italy, attending school is compulsory between 6 and 16 years, although students also have to attend other kinds of training (if they do not want to finish secondary school of second grade) until 18 [25]. Moreover, the transition from a class to the next one is not guaranteed, especially after primary school. In fact, during primary school, students are usually admitted to the next year, even if they reached the school objectives only partially. However, in exceptional cases (and upon the agreement of *all* the teachers), the child might be asked to repeat the year. Next, during secondary school of first grade, students are periodically evaluated in each subject (including behavior) through a score ranging between 0 and 10 (6 being the sufficient grade). The subsequent year’s admission is decided by the teachers (using the *majority criterion*), and the student can be admitted to the next year even if he/she did not reach all the objectives fully (i.e., at least 6 on all the subjects). In the last year (third), students must attend the state examination in order to be admitted to the next school’s level, and they are not admitted to this exam if they have had a bad grade for their behavior during the school year. Finally, students attending secondary school of second grade are still evaluated for each subject with a score ranging between 0 and 10; however, a grade under 6 for behavior does not allow them to be admitted either to the final state examination (at the fifth year) or to the next class in the previous four years [26].

From the description of the Italian school system, it is evident that students, mostly from pre-adolescence, when they enter secondary school of first grade, are faced with an ongoing evaluation of their school performance through regular written and oral exams that, jointly with their attitudes at school, are important for being admitted to the next class and the state examination. Moreover, the range of the score they can get suggests a wide range of amelioration: “good grades” range between 7 and 10 (with 10 being excellence); hence, they might feel the pressure to get the highest grade instead of a sufficient (i.e., 6) or intermediate (i.e., 7–9) grade only. Therefore, the high school dropout rate that characterizes Italian adolescents (especially students attending Professional Schools [27]) is not surprising, and it highlights the critical value of a study that addresses both the potential antecedents and outcomes of different types of Heavy Study Investment (i.e., Studyholism and Study Engagement) at an early age. This would allow for detecting the proper preventive and clinical interventions aimed at reducing Studyholism and improving Study Engagement and students’ well-being. In this vein, the present study might have vital implications from both a theoretical and a practical perspective. Besides providing insights into the appropriateness of the definition of Studyholism as an OCD-related disorder, it will also provide information about the antecedents that should be addressed for preventive and clinical purposes, which are potentially different for adolescents compared to youths. Moreover, it will inform on some different potential outcomes associated with Studyholism at different ages.

Hence, this study aims to replicate Loscalzo and Giannini’s [5] research on a sample of Italian adolescents, aiming to detect similarities and differences between the two age groups. More specifically, in line with Loscalzo and Giannini’s [5] objectives *and results*, I aim to: (i) analyze if worry is a strong predictor of Studyholism, as this might provide further evidence to the OCD-related conceptualization of Studyholism [5,14,15,16]; (ii) analyze if Studyholism has a negative effect on academic performance, psychological, and physical well-being, while Study Engagement has a positive effect on these variables; (iii) analyze if both Studyholism and Study Engagement have a negative effect on social functioning; (iv) analyze if there are differences between the two types of Studyholic (i.e., Engaged and Disengaged Studyholics) in the antecedents and outcomes analyzed.

Moreover, in line with Loscalzo and Giannini [5]’s results, I hypothesize that: (i) *Studyholism* does not predict positive emotions and GPA—or negatively predicts GPA with a low value—while it positively predicts time spent studying (generally and before exams), school dropout intention, negative emotions, general stress, sleep issues (daytime sleepiness and sleep quality impairment), relationship impairment due to study, family and friends’ complaints due to study, and quarrels at school (Loscalzo and Giannini [5] did not find support for this hypothesis concerning quarrels at university—however, they suggested that in adolescent students who attend smaller classes that allow closer relationships with both teachers and peers, this hypothesis might find support and deserve to be further analyzed); (ii) *Study Engagement* positively predicts GPA, time spent studying, and positive emotions, while it negatively predicts school dropout intention, general stress, daytime sleepiness, sleep quality impairment, and negative emotion (Loscalzo and Giannini [5] did not find support for this hypothesis with regard to the last two variables referring to their selected 0.10 cut-off value; however, both the variables had a negative *β* value of 0.08. For this reason, I maintain the hypothesis since, in adolescents, they might reach the cut-off). Moreover, Study Engagement positively predicts social impairment, as evaluated through quarrels at school, family and friends’ complaints due to study, and relationship impairment due to study; (iii) *perfectionistic strivings*, which is the perfectionistic component usually associated with positive outcomes, positively predicts Study Engagement, positive affect, study-related perfectionism, and GPA (Loscalzo and Giannini [5] did not find support for this hypothesis concerning GPA, though the *β* value was 0.08. Therefore, I maintain the hypothesis since, in adolescents, it might reach the cut-off]. Finally, I hypothesize that perfectionistic strivings do not predict Studyholism. Loscalzo and Giannini [5] did not posit a hypothesis concerning this variable since they reported that results are not consistent in the (workaholism) literature. However, they found that it does not predict Studyholism, using the same scale employed in the current study for evaluating perfectionistic strivings; (iv) *perfectionistic concerns*, which is the perfectionistic dimension usually associated with negative outcomes, does not predict Studyholism and positive affect, while it predicts, positively, study-related perfectionism, school dropout intention, negative emotions, general stress, daytime sleepiness, and sleep quality impairment. In addition, it negatively predicts Study Engagement and GPA; (v) *study-related perfectionism*, even if it is a perfectionistic facet specifically related to study, does not predict Studyholism and time spent studying (generally and before exams). However, it positively predicts Study Engagement, GPA, quarrels at school, family and friends’ complaints due to study, and relationship impairment due to study; (vi) *trait worry* positively predicts Studyholism, study-related perfectionism, negative emotions, general stress, sleep issues, and (with a low beta value) Study Engagement, while it negatively predicts positive emotions; (vii) *parents’ and teachers’ overstudying climate* positively predicts Studyholism and negatively predicts Study Engagement (Loscalzo and Giannini [5] did not find support for these hypotheses: even if the beta values were in the expected directions, they did not reach the 0.10 cut-off; however, they speculated that in younger students, there might be evidence for a greater effect of the overstudying climate on Studyholism and Study Engagement as they might be more dependent on the expectations from older significant people, such as parents and teachers). Moreover, teachers’ overstudying climate positively predicts study-related perfectionism and quarrels at school; (viii) the situational antecedent related to the *area of study* is different in the adolescent population than in college students, and it is distinguished in High School, Technical School, and Professional School. Hence, in line with Loscalzo and Giannini [1], I hypothesize that Professional and Technical schools have lower levels of Studyholism, as well as of Study Engagement, GPA, time spent studying, and relationship impairment due to study, as compared to High School students, who are usually urged towards higher academic standards. In addition, based on recent Italian data [27] that showed that Professional Schools are characterized by the highest dropout rates (i.e., 7.7% against 1.8% of High Schools and 4.3% of Technical Schools), I hypothesize that Professional School students have higher dropout intention and, therefore, higher quarrels at schools with peers/teachers compared to High School students.

In sum, I expect to find comparable results between the two age groups. However, as Loscalzo and Giannini [1] suggested both in their first theoretical paper and in their first attempt to test their model [5], some differences might be evident in younger students, especially concerning situational antecedents (in this study, overstudying climate) and outcomes (in this study, quarrels at school). In fact, younger students might be more dependent on older significant people’s expectations, such as parents and teachers. Moreover, adolescents’ classes comprise fewer students than the classes attended by college students; therefore, closer relationships with students/teachers and (negative) group dynamics might be more evident. In addition, non-college students must attend school lessons even if they do not want to, with potentially higher negative consequences on their health, as they cannot avoid school (classes’ attendance, at university, is not mandatory generally).

## 2. Materials and Methods

### 2.1. Participants

Participants included 793 Italian adolescents aged between 13 and 21 years (*M* age = 16.30 ± 1.73), balanced for gender (53% boys), and living in a city in Central Italy. The proportions of students in years 1 to 5 were 22.1%, 30.1%, 10.5%, 9.3%, 28.0%. There are three types of school for adolescents in Italy, and in this study, there are students across all of them: Professional School, 12.0%; Technical School, 50.8%; and High School, 37.2%. More specifically, four schools were contacted, and all of them agreed to participate. However, the response rates from students were different: 49.6% for the school providing one technical curriculum, one professional curriculum, and a high school curriculum; 10.6% for a school offering both a technical and a professional curriculum; 24.0% for the professional school; and 15.0% for the high school (0.8% was missing).

### 2.2. Materials

#### 2.2.1. Study-Related Perfectionism Scale (SPS)

The SPS [28] is an 11-item self-report instrument that allows for evaluating perfectionism in the school context. Although it has four scales, that is, Excessive Strivings and Concerns, Error Intolerance, Inability to Delegate, and Work Group Avoidance, the results of a second-order model support the use of a total score for both youths (Comparative Fit Index—CFI = 0.95, Root Mean Square Error of Approximation—RMSEA = 0.07) and adolescents (CFI = 0.93, RMSEA = 0.06). Moreover, the scale reliability is satisfactory for both youths (α = 0.82) and adolescents (α = 0.78) [28]. The response format is a 5-point Likert scale ranging between 1 (*Strongly Disagree*) and 5 (*Strongly Agree*). In the current sample, the alpha was 0.80.

#### 2.2.2. Penn State Worry Questionnaire (PSWQ)

The PSWQ [29] is a self-report instrument, and it measures trait worry through 16 content-free items; hence, it does not evaluate worries concerning specific periods or situations. The participants answer using a 5-point Likert scale ranging from 1 (*Strongly Disagree*) to 5 (*Strongly Agree*). The Italian PSWQ has good scale reliability, as the Cronbach’s alpha is 0.85; however, some of the reversed items are not fully satisfactory [30]. Moreover, the PSWQ has not been validated on adolescents. Hence, as a preliminary step, I performed confirmatory factor analysis (CFA) on a random subsample of 100 participants. The original scale showed an acceptable fit: CFI = 0.91; RMSEA = 0.076 (0.053–0.098), and the items load on the factor with values ranging between 0.44 and 0.81, except for item 11, which has a very low value (i.e., 0.07). However, deleting this item did not greatly improve the fit: CFI = 0.92, RMSEA = 0.078 (0.053–0.101). Hence, considering that deleting this item leads to an improvement of just 0.01 in the Cronbach’s alpha (that is, 0.91 for the 16-item total score), the original 16-item version was used in subsequent analyses.

#### 2.2.3. Overstudy Climate Scale-Revised (OCS-R)

The OCS-R [31] is a 12-item self-report scale that evaluates parents’ overstudying climate (P-OSC) and teachers’ overstudying climate (T-OSC), through six items each. The response format is a 5-point Likert scale ranging between 1 (*Strongly Disagree*) and 5 (*Strongly Agree*). It is a shorter version as compared to the Overstudy Climate Scale (OCS) [32] used by Loscalzo and Giannini [5], and it has just one scale for T-OSC, instead of two sub-scales. The OCS-R has good psychometric properties on both youths and adolescents. Concerning adolescents, the fit of the 2-factor model is good: CFI = 0.96, RMSEA = 0.048 (0.027–0.067), as well as the internal reliability: P-OSC, α = 0.81; T-OSC, α = 0.74 [31]. In the current sample, the alpha value was 0.86 for P-OSC and 0.79 for T-OSC.

#### 2.2.4. Short Almost Perfect Scale (SAPS)

The SAPS [33] is an 8-item self-report instrument that evaluates both perfectionistic strivings and concerns through its Standards and Discrepancy subscales. The response format is a 7-point Likert scale ranging from 1 (*Strongly Disagree*) to 7 (*Strongly Agree*). We administered the Italian version of the SAPS [34,35] that foresees the use of six items, three for each scale (instead of eight), for both college students—CFI = 0.95; RMSEA = 0.10; Standards, α = 0.83; Discrepancy, α = 0.68 [34]—and adolescents—CFI = 0.94; RMSEA = 0.088 (0.053–0.126); latent variables’ internal reliability: Standards, 0.76 (95% CI: 0.71–0.81); Discrepancy, 0.70 (95% CI: 0.63–0.77)—[28]. In the current sample, the alpha was 0.80 for Standards and 0.65 for Discrepancy.

#### 2.2.5. Studyholism Inventory (SI-10)

The SI-10 [11] is a 10-item self-report made up of two scales, namely Studyholism and Study Engagement, and comprising a head-sheet to assess some study habits, such as studying on the weekend, time spent studying, and GPA. The response format is a 5-point Likert scale ranging between 1 (*Strongly Disagree*) and 5 (*Strongly Agree*). The SI-10 is currently available in Italian, Polish, Croatian, Spanish, and English. For this study, we used the Italian version [6,11], which also has good psychometric properties on Italian pre-adolescents and adolescents—for the adolescent sample, the fit indexes are good: CFI = 0.97, RMSEA = 0.059 (0.039–0.080), and the scale reliability is satisfactory: Studyholism, α = 0.78; Study Engagement, α = 0.79 [13]. In the current sample, the alpha was 0.83 for Studyholism and 0.82 for Study Engagement.

#### 2.2.6. Mini Sleep Questionnaire (MSQ)

The MSQ [36] comprises ten items and two subscales that allow for evaluating sleep quality impairment and daytime sleepiness. The participants answer through a 7-point Likert scale ranging between 1 (*Never*) and 7 (*Always*). The Italian version [37] does not include one item for scoring purposes (GFI = 0.95, RMSEA = 0.086; Cronbach’s alpha is 0.75 for both the scales) and it has also been used in adolescence [38]. However, Tonetti et al. [38] did not perform CFA. Therefore, I conducted a preliminary CFA on a random subsample of 100 participants, finding an acceptable fit: CFI = 0.90; RMSEA = 0.10 (0.058–0.139). The factor loadings are good, ranging between 0.38 and 0.75, and the scales have average Cronbach’s alpha values (0.75 for Daytime Sleepiness, 0.76 for Sleep Quality).

#### 2.2.7. Study-Relationships Conflict Scale (SRCS)

The SRCS [39] is a 9-item self-report instrument that evaluates quarrels at school (QS), relationship impairment (RI), and family and friends’ complaints (FFC). The participants answer using a 5-point Likert scale ranging between 1 (*Strongly Disagree*) and 5 (*Strongly Agree*). This scale has good psychometric properties in the original version validated on college students: CFI = 0.98; RMSEA = 0.038; QS, α = 0.67, RI, α = 0.63; FFC, α = 0.64 [30]. Moreover, as highlighted by the preliminary CFA conducted on a random subsample of 100 participants, it might be used with adolescents as well: CFI = 0.95; RMSEA = 0.065 (0.000–0.111). Regarding internal reliability, the alpha values were similar to the ones found by Loscalzo and Giannini [39]: QS = 0.73; RI = 0.64; FFC = 0.67.

#### 2.2.8. Depression Anxiety Stress Scales-21 (DASS-21)

The DASS-21 [40] is a 21-item self-report scale that allows for evaluating depression, anxiety, and stress, and (using the total score) general stress. The participants answer using a 4-point Likert scale ranging between 0 (*Did not apply to me at all*—*Never*) and 3 (*Applied to me very much, or most of the time*—*Almost always*). The Italian version has good psychometric properties: CFI = 0.98; RMSEA = 0.038; General Stress, α = 0.90. [41]. However, it has not been validated on adolescents. Hence, also in this case, I performed a preliminary CFA on a random subsample of participants. Since, in this study, only the total score will be used (in line with Loscalzo and Giannini [5]), I tested the bi-factor model of Bottesi et al. [41], finding acceptable fit indexes: CFI = 0.90; RMSEA = 0.087 (0.070–0.103). The alpha value for the total score was even higher than the one reported by Bottesi et al. (2015), as it is 0.95.

#### 2.2.9. Positive and Negative Affect Schedule (PANAS)

The PANAS [42] is a 20-item self-report instrument for the evaluation of positive and negative affect. The response format is a 5-point Likert scale ranging from 1 (*Very slightly* or *not at all*) to 5 (*Extremely*). It is available in two versions, which differ in the instruction only, as they can refer to positive and negative affect as a trait or as a state. In this study, in line with Loscalzo and Giannini [5], I used the state version. The Italian version [43] has satisfactory psychometric properties (CFI = 0.80, RMSEA = 0.09, Cronbach’s alpha is 0.85 for state negative affect and 0.83 for state positive affect), and it has been previously used with Italian adolescents [44,45]. In the current sample, the alpha values for positive and negative affect were, respectively, 0.87 and 0.92.

#### 2.2.10. Dropout Intention

Dropout intention was evaluated through the three items by Hardre and Reeve [46], which were translated into Italian by Alivernini and Lucidi [47]. The response format is a 5-point Likert scale ranging from 1 (*Never*) to 5 (*Very often*). In the current sample, the alpha value was 0.82.

### 2.3. Procedure

After obtaining approval from the Ethical Committee of the University of Florence, it has been looked for school heads who might have been interested in collaborating in the research by allowing for collecting data among students. Hence, four schools were contacted to ask for their authorization to collect data in their schools. They were selected based on the curricula they offered, since I aimed to have participants across all the three Italian types of school (i.e., High School, Technical School, Professional School). Next, informed consent, signed by both the participants and their parents, was gathered. In this document, parents were required to provide an e-mail address to receive the online questionnaire that the son/daughter filled out. The online questionnaire comprised the instruments previously described and a preliminary section for demographic data (e.g., gender, age).

### 2.4. Data Analysis

I performed the analyses through SPSS.26 (IBM, Chicago, IL, USA) and AMOS.20 (IBM, Chicago, IL, USA).

First, I used linear interpolation to estimate the values for missing cases present in the following variables: time spent studying daily (8 missing cases; 1%) and time spent studying before exams (5 missing cases; 0.63%). Next, I analyzed the variables’ descriptive statistics (including skewness and kurtosis), and I looked for potential outliers. Then, I calculated the zero-order correlations of the variables included in the model, and I performed a structural equation model (SEM), more specifically a path analysis (maximum likelihood estimate method), to analyze the direct effects of the antecedents on Studyholism, Study Engagement, and on other Studyholism antecedents (e.g., worry on study-related perfectionism), and the effects of Studyholism and Study Engagement on academic, psychological, and physical outcomes (as well as of some antecedents on the outcomes). However, since the multivariate normality assumption was not met (as evaluated trough Mardia’s test), I also used the bootstrapping procedure to evaluate the 95% bias-corrected confidence intervals, which is the procedure suggested by Byrne [48] to deal with data that do not respect the multivariate normality assumption through AMOS. To evaluate the fit of the model, I referred to the cut-off values provided by Byrne [48], Hu and Bentler [49], and Reeve et al. [50]. In line with Loscalzo and Giannini [5], *p* < 0.05 was considered statistically significant, and *β* = ±0.10 was the cut-off value for supporting a significant value.

Finally, I analyzed the prevalence of high/low Studyholism/Study Engagement and the prevalence of the four types of student (i.e., Engaged Studyholics, Disengaged Studyholics, Engaged students, Detached students), as well as if there were differences between Disengaged and Engaged Studyholics in the antecedents and outcomes through Mann–Whitney tests. The number of participants belonging to the two types of Studyholic did not allow for performing parametric analyses. The two types of Studyholic were created referring to the SI-10 cut-off values for Italian adolescents [13].

## 3. Results

### 3.1. Structural Equation Model

Table 1 shows the descriptive statistics of the variables analyzed in the SEM model, while Table 2 shows the zero-order correlations of the study variables. Table 1 highlights that most of the variables have a normal distribution, with a few having a value just slightly higher than one for skewness and kurtosis. However, the variable regarding the hours of study per day generally has a kurtosis value of about 4. Although, I retained this variable in the model, in line with Loscalzo and Giannini [5]. In fact, they found a variable that was not normally distributed, but they retained it in the model as it was not a predictor. In addition, Bentler [51] suggested that values higher than five indicate that data are not normally distributed, and the present value is lower than this cut-off. Finally, the outlier analyses suggest two potential outliers; however, a closer inspection of the data suggested not removing them. More specifically, the potential outlier for time spent studying generally scores 8, while the closer subject scores 7; also, the 5% triggered mean (2.40) is very similar to the regular mean (2.44). In the same line, the potential outlier for time spent studying before exams scores 12, while the subsequent subject scores 10. However, also in this case, the 5% triggered mean (3.06) is similar to the regular mean (3.12).

However, Mardia’s normalized estimate of multivariate kurtosis suggests that the data do not respect the multivariate normality assumption, having a value of 73.74. Therefore, aiming to replicate the model tested by Loscalzo and Giannini [5] on Italian college students, I performed a path analysis (maximum likelihood estimate method), but I also applied the bootstrapping procedure (requesting 500 bootstrap samples and 95% bias-corrected confidence intervals).

The model showed a good fit to the data: CFI = 0.94; NFI = 0.92; RMSEA = 0.060 (C.I. 90% = 0.055–0.066). The χ^2^/df value (χ^2^ = 477.096, *df* = 123, *p* < 0.001, χ^2^/df *=* 3.88) and the Bollen-Stine bootstrap (*p* = 0.002) are not satisfactory, although this is in line with the fact that the *χ^2^* index is influenced by the sample size [52]. Table 3 shows the variance explained by the predictors of each dependent variable, the standardized path estimates (maximum likelihood estimation) with their bias-corrected confidence intervals (bootstrapping procedure), and if the results are in line (or not) with those of college students [5]. As shown in Table 3, the bootstrapping procedure results confirm the results obtained by applying the maximum likelihood estimation. The only difference concerns the effect of Professional School on Studyholism, since the bias-corrected confidence interval reports that it is not significant. However, this path’s beta value (*β* = –0.06) did not reach the selected 0.10 cut-off value (and its *p* value was 0.04) also within the maximum likelihood estimation method. Hence, the difference concerning this path does not affect the results arising from the analyses.

Concerning the main results displayed by the path analysis, I found equivalent results to Loscalzo and Giannini’s [5] findings. More specifically, the variables explain 48% of the variance in Studyholism, with worry being the strongest predictor (*β* = 0.62, *p* < 0.001). Instead, perfectionism is not a predictor of Studyholism in any of the three components included in the model. Regarding Study Engagement, the model explains 29% of its variance, and perfectionistic strivings is the strongest predictor (*β* = 0.41, *p* < 0.001). Moreover, general stress is the outcome variable with the highest percentage of variance explained. However, in adolescents, Studyholism predicts it with a lower beta value (β = 0.13) compared to college students (*β* = 0.25). Finally, the model explains a lower percentage of dropout intention’s variance in adolescents (18% against 27%), although Studyholism and Study Engagement still predict it in different directions, with Studyholism being a positive predictor of dropout intention.

### 3.2. Differences between Engaged and Disengaged Studyholics

First, using Loscalzo et al. [13]’s cut-off values for defining high/low Studyholism/Study Engagement in adolescents, I analyzed the prevalence of the four types of student suggested by Loscalzo and Giannini [1]. I found that, in this sample, there are 135 (17.0%) students with high Studyholism and 91 (11.5%) students with low Studyholism; moreover, there are 90 (11.3%) adolescents with high Study Engagement and 127 (16.0%) adolescents with low Study Engagement. Concerning the types of student, these are the percentages: Engaged Studyholics, 3.4% (*n* = 27); Disengaged Studyholics, 1.8% (*n* = 14); Engaged students, 0.1% (*n* = 1); and Detached students, 9.1% (*n* = 72).

Next, I performed Mann–Whitney tests to evaluate differences in the antecedents and outcomes between Disengaged and Engaged Studyholics (Table 4 shows the results of these analyses). Regarding the antecedents, there is a statistically significant difference between Engaged and Disengaged Studyholics in perfectionistic strivings and study-related perfectionism, with Engaged Studyholics scoring higher. There is no difference in perfectionistic concerns, worry, and overstudy climate. Regarding outcomes, Engaged Studyholics reported statistically significantly greater time spent studying generally, higher GPA, and more family and friends’ complaints due to studying. However, they reported lower daytime sleepiness, quarrels at school, negative affect, and dropout intention than Disengaged Studyholics. There is no difference in sleep quality impairment, relationship impairment due to study, positive affect, general stress, and time spent studying before exams.

## 4. Discussion

This study aims to deepen the analysis of Studyholism in adolescence by replicating Loscalzo and Giannini’s [5] study regarding potential antecedents and outcomes of Studyholism (and Study Engagement), as well as regarding the differences between Engaged and Disengaged Studyholics.

The path analysis’ findings showed, as expected, comparable results to those found by Loscalzo and Giannini [5] on college students, even if with a few differences.

Regarding individual antecedents, in line with Loscalzo and Giannini [5], Studyholism is strongly predicted by (trait) worry. Moreover, perfectionism does not predict Studyholism in any of the three components included in the model, namely perfectionistic strivings, perfectionistic concerns, and study-related perfectionism. Regarding Study Engagement, in line with Loscalzo and Giannini [5], it is positively predicted by perfectionistic strivings and study-related perfectionism, while perfectionistic concerns negatively predict it. In contrast with the college students’ sample, and in line with my hypothesis, worry also positively predicts (with a low value) Study Engagement. However, in the adolescent sample, the *β* value is slightly higher than the selected 0.10 cut-off, while in college students, it is slightly lower than the cut-off (0.07). Hence, the results are actually quite similar since the beta values are positive and low in both of the Italian samples. In fact, I based my hypothesis regarding the predictive value of trait worry on Study Engagement on the value found by Loscalzo and Giannini [5].

Concerning situational antecedents, in line with Loscalzo and Giannini’s results [5], and in contrast with my hypothesis, parents’ overstudying climate does not predict Studyholism and Study Engagement. Regarding teachers’ overstudying climate, it does not predict Study Engagement, like for college students (and, again, in contrast with my hypothesis), while it positively predicts Studyholism. However, in this case, the difference with Loscalzo and Giannini [5] is not critical. In fact, the *β* value for the adolescent sample barely reaches the 0.10 cut-off. Moreover, in the college sample, where the scale used for evaluating the teachers’ overstudying climate comprised two subscales (i.e., overt comments about the performance and pressure to study hard), the *β* values were 0.02 and 0.08, respectively. Finally, regarding the type of school, it predicts neither Studyholism nor Study Engagement (against my hypothesis). Even though this antecedent was evaluated in college students using the major of study, hence not allowing a direct comparison, the results might be considered equivalent since the study area also does not predict the two types of Heavy Study Investment in college students.

In sum, *the results regarding the individual and situational antecedents in Italian youths and adolescents are comparable*. Therefore, from a theoretical point of view, I found further support for Loscalzo and Giannini’s [5,18] speculation that workaholism (which is usually associated with perfectionistic strivings and concerns [17]) and Studyholism might differ in their antecedents, and hence deserve to be studied as two different constructs. However, this is just the second study analyzing the relationship between perfectionism and Studyholism. Hence, other studies should be performed, possibly including other perfectionism scales, aiming to shed light on the role of perfectionism, and its different facets, as a correlate of Studyholism (the correlation analysis, in fact, showed positive values of correlation ranging between 0.17 and 0.34). Moreover, I found further support for the definition of Studyholism as an OCD-related disorder. Concerning preventive and clinical interventions, trait worry is still the variable to be primarily targeted to prevent/reduce Studyholism, while perfectionism and overstudying climate do not seem to be valuable as targets with these purposes. Moreover, since the type of school does not predict Studyholism, it has been confirmed that preventive interventions should be implemented across all the types of school (in college students, across all majors of study).

Regarding Studyholism (and Study Engagement) academic outcomes, the results confirm that Studyholism does not predict GPA (even though the beta value is negative, it is under the 0.10 cut-off), while Study Engagement is a positive predictor of it. In addition, dropout intention is still positively predicted by Studyholism and negatively predicted by Study Engagement. Moreover, both Studyholism and Study Engagement positively predict the time spent studying generally and before exams. However, compared to Loscalzo and Giannini [5], Studyholism has a higher *β* value for time spent studying both generally and before exams; moreover, the time spent studying before exams is predicted at the greatest extent by Studyholism (instead of by Study Engagement). Hence, these results suggest that *Studyholism, in adolescence, plays a stronger role in the time spent studying, especially before exams*. However, despite this greater time investment in studying associated with Studyholism in adolescence, the academic impairment associated with Studyholism is still confirmed (and the academic success that characterizes Study Engagement instead). Therefore, also in this case, the preventive interventions suggested by Loscalzo and Giannini [5] for improving academic success are valuable for adolescents as well. Since Studyholism is associated with higher time spent studying (like Study Engagement), but not with higher GPA and lower dropout intention, interventions should fuel Study Engagement, reduce Studyholism, and train students to organize their time investment in study fruitfully.

Concerning psychological outcomes, the results are again similar to Loscalzo and Giannini [5]. Studyholism does not predict positive affect, while it is a positive predictor of negative affect and general stress. However, in college students, the effect of Studyholism on negative affect and general stress is higher than in adolescents. Thus, *the psychological impairment associated with Studyholism is higher in youths compared to adolescents*. Regarding Study Engagement, it is still a positive predictor of positive affect (even if with a lower value) and a negative predictor of general stress. However, as hypothesized, Study Engagement in adolescents is also a negative predictor of negative affect (in college students, it is a negative predictor, but the beta value does not reach the 0.10 cut-off). Moreover, it has a higher *β* value than Studyholism for both negative affect and stress (in college students, instead, Studyholism has a higher *β* value than Study Engagement for these psychological variables). Hence, compared to college students’ results [5], *Studyholism plays a weaker role in psychological impairment in adolescence, while Study Engagement plays a more substantial role in favoring psychological well-being.* Therefore, I speculate that it is critical to implement preventive and clinical interventions aimed at fostering Study Engagement and reducing Studyholism at an early age, possibly during pre-adolescence (or even during primary school), aiming to avoid the onset or maintenance of problematic overstudying at an older age, and hence a higher psychological impairment. In line with this, it is critical to validate an adapted version of the Studyholism Inventory (SI-10) [11] that might be used with children and create a parent-report version applicable from the first years of primary school. In addition, this implies a critical theoretical implication: as suggested by Loscalzo and Giannini [1], Studyholism might arise in pre-adolescence due to the increase in the time and effort required by school during this age; however, I speculate that some early indicators (or, maybe, even the onset in some cases) might be evident earlier, namely during primary school (i.e., childhood), and they should be detected (and managed) to prevent the chronicization of Studyholism at an older age.

Regarding physical outcomes, Studyholism is confirmed to be, also in adolescents, a positive predictor of both sleep quality impairment and daytime sleepiness, while Study Engagement is a negative predictor of these two variables. In Loscalzo and Giannini’s results [5], Study Engagement does not reach the 0.10 cut-off value for sleep quality impairment; however, it was a negative predictor (–0.08). Hence, also in this case, the difference is minimal, and *the negative effect of Studyholism on physical well-being (and the protective factor of Study Engagement) have received further support*, providing further evidence for the crucial importance of addressing Studyholism at an early age.

Finally, the main differences between youths and adolescents are in regard to social outcomes. Besides the similarity with regard to relationship impairment due to study, which is positively predicted by both Studyholism and Study Engagement in both youths and adolescents, there are some differences concerning quarrels at school and family and friends’ complaints due to study. More specifically, quarrels at school are not predicted by Studyholism, in contrast with my hypothesis and in line with Loscalzo and Giannini [5]; however, in line with my hypothesis, they are negatively predicted by Study Engagement, and the *β* value is good (–0.20), while in college students, the *β* value did not reach the 0.10 cut-off, being –0.08. In addition, in adolescents, Study Engagement is still a positive predictor of family and friends’ complaints due to overstudying; however, in contrast with Loscalzo and Giannini [5] and my hypothesis, Studyholism does not predict it. Hence, *in adolescents, there is a higher protective effect of Study Engagement on a situational social outcome, namely quarrels at school*, providing support for the importance of fueling this positive attitude toward study to obtain a better school climate. However, in adolescents, Study Engagement is associated with both family and friends’ complaints due to study and relationship impairment, while *Studyholism is associated with higher relationship impairment only*. Though, Study Engagement has a stronger predictive effect on family and friends’ complaints than Studyholism in both the adolescents’ and youths’ sample. In sum, *in line with the results regarding psychological impairment, which suggested that Studyholism plays a weaker role in adolescence, social impairment also seems to be predicted to a lesser extent by Studyholism during adolescence*. Hence, this further confirms the need to implement preventive and clinical interventions aimed at fostering Study Engagement and reducing Studyholism at an early age to avoid higher psychological and social impairment when students are older and, therefore, it also implies—from a theoretical point of view—the need to analyze Studyholism’s features in childhood and pre-adolescence. Moreover, the positive association between Study Engagement and social impairment further supports Loscalzo and Giannini’s [5] suggestion to develop interventions to foster students’ well-being addressed both to Engaged students and Studyholics. In this vein, it would be vital to train students to appropriately manage the time devoted to study and social relations, for example, by increasing their time management skills.

Regarding the other relationships that were analyzed among the variables included in the path analysis model, in line with the hypotheses that I grounded in Loscalzo and Giannini [5]’s results, the differences are: (i) teachers’ overstudying climate does not predict study-related perfectionism in adolescence—however, in college students, only the scale regarding overt comments concerning the performance was a predictor; (ii) perfectionistic strivings, in adolescence, is a positive predictor of GPA; (iii) perfectionistic concerns is not a predictor of dropout intention; (iv) worry does not predict (negatively) positive affect; (v) study-related perfectionism does not predict quarrels at school, since the beta value is under the 0.10 selected cut-off—however, in college students, the beta value is slightly higher than 0.10 (i.e., 0.13).

However, despite these few differences, there are no differences concerning the implications that arose when analyzing these other relationships for preventive interventions. In fact, even if perfectionistic concerns do not predict higher dropout intention, it is still a negative predictor of Study Engagement and GPA, and a positive predictor of negative affect, general stress, sleep quality impairment, and daytime sleepiness. Therefore, also in adolescents, it is an individual variable to be reduced to favor students’ well-being and Study Engagement. Regarding the domain-specific type of perfectionism, which is study-related perfectionism, it is still a positive predictor of Study Engagement and (to a lesser extent compared to youths) GPA; however, it is also still associated with perfectionistic concerns (as this form of perfectionism predicts it), family and friends’ complaints, and relationship impairment due to study. Hence, this study confirmed that, both in adolescents and youths, study-related perfectionism should not be fostered as it is associated with academic success, but also with negative outcomes, and especially with social impairment. Finally, regarding the overstudying climate, in adolescence, teachers’ overstudying climate is still a predictor of aggressive behaviors at school, in the form of quarrels. Thus, this suggests that teachers, even if they do not play a strong role in fostering Studyholism (and in reducing Study Engagement), can increase quarrels between students and between students and teachers, hence affecting the class climate. Hence, in line with Loscalzo and Giannini [5], it seems crucial to implement training interventions for teachers to make them conscious of the negative effect they can have on the class climate by prompting overstudying behaviors.

Concerning the relationships between the type of school and some of the other variables included in the model, I found support for the hypothesis that Professional and Technical Schools negatively predict GPA. Hence, students of these two types of schools have a lower GPA than High School students. In line with this, as hypothesized, they are also characterized by less time spent studying generally and before exams. However, contrary to my hypothesis, High School students do not have higher relationship impairment due to study than the other students. Finally, in line with my hypothesis, Professional Schools students have higher dropout intentions and quarrels at school than High School students. Therefore, interventions to improve class climate and reduce dropout intentions are essential for Professional Schools’ students.

Regarding the differences between Engaged and Disengaged Studyholics in the antecedents and outcomes, Table 5 shows both the similarities and differences with Loscalzo and Giannini’s [5] results.

I found that in adolescents (in line with Loscalzo and Giannini, [5]), Engaged Studyholics have higher perfectionistic strivings and study-related perfectionism than Disengaged Studyholics. However, there is no difference concerning perfectionistic concerns (while, in youths, this perfectionistic component was higher in Disengaged Studyholics). Regarding the other antecedents (worry and overstudy climate), there is no difference between the two types of Studyholics, as for the youths’ sample. Regarding physical outcomes, Disengaged Studyholics experience more daytime sleepiness, while in youths there was no difference on the two sleep-related variables. For psychological outcomes, Disengaged Studyholics are still more impaired. However, in this study, the difference is statistically significant only for negative affect, while in youths, it was significant also for positive affect (though the median value is higher for Engaged Studyholics in adolescents too). For social outcomes, Engaged Studyholics are still more impaired since they have higher family and friends’ complaints due to study; however, there is no difference in relationship impairment due to study (though, also in this case, the median value is higher for Engaged Studyholics, as for college students). Moreover, in adolescents only, Engaged Studyholics cause a lower level of impairment in the school context than Disengaged Studyholics, since lower levels of quarrels at schools characterize them. Hence, this result confirms Loscalzo and Giannini’s [1] speculation that, in younger students, the effect of Heavy Study Investment on the class climate might be greater due to the smaller size of the classes. Finally, Disengaged Studyholics are still more impaired concerning academic outcomes, as having a lower GPA and higher dropout intention than Engaged Studyholics, who are still characterized by a higher time spent studying generally (though they do not differ in time spent studying before exams). In sum, despite a few differences compared to youths, *the higher impairment in the affective and academic areas for Disengaged Studyholics is confirmed, with the addition of a higher impairment in daytime sleepiness* (which is a physical outcome). *In contrast, Engaged Studyholics are more impaired in the social area (even if lower situational social downsides characterize them)*. In addition, there are variables in which the two types of Studyholics do not differ: general stress and sleep quality (like in Loscalzo and Giannini, [5]), and positive affect, relationship impairment due to study, and time spent studying before exams. Regarding the antecedents, in line with youths, there is no difference in worry and overstudying climate. In sum, as previously found by Loscalzo and Giannini [5], it is critical to differentiate between Disengaged and Engaged Studyholism, since there might be some different relationships with the same antecedents and outcomes under evaluation. Moreover, Disengaged Studyholics are not more impaired than Engaged Studyholics across all the areas.

Among the limitations of this study, the sample—even if balanced for gender and heterogeneous concerning the year of study and type of school—is made up of students from Central Italy only and from four schools only; therefore, this might impair the generalizability of the results. Moreover, GPA was gathered through a self-reported question; hence, it is possible that some students did not report their real GPA. In addition, the instrument used for evaluating perfectionistic strivings and concerns, namely the Short Almost-Perfect Scale (SAPS) [33], does not have fully satisfactory psychometric properties in its Italian version [34]. Hence, future studies analyzing the role of perfectionism on Studyholism using other instruments will be critical to shed light on the relationships between perfectionism facets and Studyholism. Finally, in this study, I could not perform parametric analyses for evaluating the differences between the types of student since the number of students belonging to each type did not allow such analyses. For this reason, I focused on comparing Engaged and Disengaged Studyholics only, while Loscalzo and Giannini [5] performed parametric analyses and compared all four types of student on each variable.

Besides these limitations, the present study has the merit of having analyzed many potential antecedents and outcomes of Studyholism by comparing previous results found for college students (Loscalzo and Giannini, [5]); hence, it provided further knowledge on this new potential clinical condition. Moreover, it is just the second research conducted on Studyholism, and to the best of my knowledge, it is the first study analyzing the correlates of problematic overstudying on adolescents, with important implications for preventive and clinical interventions aimed at reducing Studyholism and favoring Study Engagement, students’ well-being, and academic success. More specifically, this study’s results further support Loscalzo and Giannini’s [5] suggestions about targeting trait worry for preventing (or reducing) Studyholism while trying to reduce both perfectionistic concerns and study-related perfectionism to improve students’ well-being and academic success. Moreover, Engaged students should receive preventive interventions to improve their time management skills and, hence, be successful in balancing study duties and social/leisure activities. From a clinical perspective, it is critical to tailor the intervention to the student by distinguishing between Engaged and Disengaged Studyholics. Finally, preventive and clinical interventions should be addressed across all school types, since this is not a situational antecedent of Studyholism (like the major of study, which does not predict Studyholism in youths).

Based on the present study’s findings, I also stress the importance of implementing preventive interventions at an early age, even during primary school, as this research highlights that, in adolescents, Studyholism plays a weaker role in psychological and social impairment compared to youths. Hence, detecting early risk indicators and addressing Studyholism during childhood or pre-adolescence might help to avoid the onset or chronicization of Studyholism in adolescents and youths. Future studies employing longitudinal analysis would be critical in providing support to this assertion; moreover, the validation of a version of the Studyholism Inventory (SI-10) [11] that might be administered to children and their parents is recommended.

## 5. Conclusions

This study is the first addressing problematic overstudying in adolescence, besides being just the second study analyzing potential antecedents and outcomes of Studyholism.

In sum, from a theoretical point of view, it provides further support to Loscalzo and Giannini’s [1] conceptualization of problematic overstudying as an obsessive-compulsive related disorder (hence, an internalizing disorder) instead of as an externalizing disorder/behavioral addiction. Moreover, it also confirmed the critical importance of distinguishing between Engaged and Disengaged Studyholics, and to detect the main area of functional impairment. Finally, the finding that Studyholism seems to have a weaker role in psychological and social impairment during adolescence—and that its adverse effects become more evident in youths—suggests the need to look for early indicators of Studyholism since childhood to prevent, as early as possible, the development of Studyholism and its maintenance (with higher functional impairment) at an older age. Moreover, the finding that situational antecedents do not play a substantial role in fueling Studyholism, while trait worry is a strong predictor, suggests that studies aimed at detecting potential early indicators of Studyholism should focus on individual variables that the literature showed to be precursors of other widely analyzed internalizing disorders.

Concerning practical implications, from a clinical perspective, it would be important to target trait worry for reducing Studyholism; hence, as previously suggested by Loscalzo and Giannini [5], it could be helpful to implement programs that proved to be effective in reducing worry. In addition, due to some differences in the antecedents and outcomes between Disengaged and Engaged Studyholics, it is critical to distinguish between them to tailor the intervention to the student. From a preventive perspective, instead, the results support the need for interventions aimed at favoring students’ academic success and well-being both in Engaged students and in Studyholics. More specifically, perfectionistic concerns and study-related perfectionism are important to address in order to decrease them. In addition, time management skills should be enhanced in students. Therefore, I suggest implementing school-based preventive interventions that, through circle times and play therapy techniques, might make students aware of the negative outcomes associated with these perfectionism variables and let them experience the positive effect of social interactions. Consequently, they will probably be more intrinsically motivated to find a proper balance between time devoted to study and social activities, and hence they will also probably improve both their academic performance and well-being. Moreover, by involving teachers in these activities, it might favor a better school climate, as teachers might be prompted to feel the importance of other activities, besides studying, for the adolescents. Therefore, teachers will probably be more prone to avoid implementing an overstudying climate that causes, in particular, quarrels at school (and hence damages the school climate). Finally, besides realizing these interventions across all the school types, it is critical to prevent Studyholism as soon as possible during school years, such as during childhood or pre-adolescence, also by detecting early indicators of Studyholism.

In conclusion, I strongly encourage further study on Studyholism to deepen the analysis regarding its real internalizing/externalizing nature since the current literature about problematic overstudying is still too scant and reaching any firm conclusion about its nature seems premature. Moreover, it would be useful to analyze the effectiveness of interventions based on the suggestions arisen from this and Loscalzo and Giannini’s [5] study. Finally, longitudinal studies (from childhood) are recommended to analyze Studyholism and Study Engagement trajectories during different ages and for detecting early indicators of Studyholism.

## Figures and Tables

**Table 1:** Descriptive statistics of the variables in the model (*n* = 793).

Variable	Range	M(DS)	Skeweness	Kurtosis
Studyholism	4–20	12.57 (4.00)	–0.18	–0.75
Study Engagement	4–20	13.19 (3.61)	–0.33	–0.27
Perfectionistic Concerns	3–21	12.65 (3.99)	0.05	–0.50
Perfectionistic Strivings	3–21	15.05 (3.85)	–0.43	–0.29
Study-related Perfectionism	11–53	25.19 (7.68)	0.49	–0.14
Worry	16–78	47.48 (13.50)	0.03	–0.65
Parents’ Overstudy Climate	6–30	19.25 (5.58)	–0.09	–0.67
Teachers’ Overstudy Climate	6–30	18.95 (4.87)	0.01	–0.33
Grade Point Average *	2–10	6.82 (.97)	–0.49	1.60
Hours per day of study—generally	0–11	2.46 (1.32)	1.08	4.12
Hours per day of study—before exams	0–12	3.12 (1.56)	0.69	1.18
Dropout Intention	3–15	5.91 (3.16)	1.11	0.48
Positive Affect	10–50	27.95 (8.47)	–0.10	–0.41
Negative Affect	10–50	19.85 (9.27)	0.86	–0.07
General Stress	0–62	21.03 (13.85)	0.61	–0.32
Sleep Quality Impairment	5–33	15.22 (6.34)	0.43	–0.50
Daytime Sleepiness	4–28	15.97 (5.52)	0.06	–0.75
Quarrels at School	3–15	5.28 (2.55)	1.17	0.87
Family and Friends’ Complaints	3–14	4.88 (2.38)	1.28	0.87
Relationship Impairment	3–15	5.22 (2.59)	1.13	0.50

*Note*. * = Italian GPAs range between 0 and 10; 6 stands for a sufficient evaluation.

**Table 2:** Zero-order correlations for study variables (*n* = 793).

Variable	1	2	3	4	5	6	7	8	9	10	11	12	13	14	15	16	17	18	19	20
1.PC	-																			
2.PS	0.17 ***	-																		
3.SPS	0.29 ***	0.34 ***	-																	
4.PSWQ	0.40 ***	0.18 ***	0.36 ***	-																
5.P-OCS	0.30 ***	–0.001	0.09 **	0.15 ***	-															
6.T-OSC	0.23 ***	0.08 *	0.11 **	0.22 ***	0.27 ***	-														
7.SH	0.34 ***	0.17 ***	0.28 ***	0.68 ***	0.17 ***	0.26 ***	-													
8.SE	–0.05	0.46 ***	0.33 ***	0.17 ***	–0.01	0.03	0.27 ***	-												
9.GPA	–0.18 ***	0.34 ***	0.20 ***	0.04	–0.15 ***	–0.06	0.03	0.41 ***	-											
10.H-Gen	0.09 *	0.20 ***	0.20 ***	0.27 ***	–0.03	0.03	0.35 ***	0.43 ***	0.19 ***	-										
12.H-Exams	0.09 **	0.21 ***	0.14 ***	0.25 ***	0.05	0.01	0.36 ***	0.31 ***	0.27 ***	0.54 ***	-									
12.Drop Int.	0.20 ***	–0.14 ***	–0.03	0.17 ***	0.08 *	0.15 ***	0.24 ***	–0.22 ***	–0.34 ***	–0.10 **	–0.07	-								
13.PANAS+	–0.04	0.31 ***	0.04	–0.02	0.04	0.08 *	0.01	0.27 ***	0.11 **	0.11 **	0.04	0.02	-							
14.PANAS−	0.33 ***	–0.04	0.12 **	0.39 ***	0.13 ***	0.19 ***	0.31 ***	–0.07 *	–0.11 ***	0.07	0.05	0.32 ***	0.22 ***	-						
15.DASS-21	0.38 ***	0.02	0.21 ***	0.57 ***	0.18 ***	0.22 ***	0.44 ***	–0.07	–0.10 **	0.06	0.05	0.39 ***	0.04	0.62 ***	-					
16.MSQ-SQ	0.31 ***	0.03	0.12 ***	0.40 ***	0.15 ***	0.23 ***	0.33 ***	−0.07 *	–0.17 ***	0.02	0.03	0.34 ***	0.02	0.40 ***	0.61 ***	-				
17.MSQ-DS	0.31 ***	0.06	0.05	0.39 ***	0.13 ***	0.24 ***	0.37 ***	–0.07	–0.11 ***	0.07 *	0.08 *	0.37 ***	0.03	0.40 ***	0.57 ***	0.61 ***	-			
18.SRCS-Q	0.20 ***	–0.05	0.03	0.12 ***	0.07	0.23 ***	0.07 ***	–0.16 ***	–0.22 **	–0.16 ***	–0.11 ***	0.35 ***	0.05	0.34 ***	0.36 ***	0.33 ***	0.32 ***	-		
19.SRCS-C	–0.03	0.23 ***	0.34 ***	0.17 ***	–0.05	0.08 *	0.13 ***	0.39 ***	0.32 ***	0.30 ***	0.15 ***	–0.09 *	0.06	0.05	0.12 ***	0.07	0.03	0.11 ***	-	
20.SRCS-RI	0.16 ***	0.18 ***	0.32 ***	0.37 ***	0.10 **	0.14 ***	0.38 ***	0.31 ***	0.11 **	0.40 ***	0.31 ***	0.09 *	0.02	0.20 ***	0.30 ***	0.21 ***	0.19 ***	0.14 ***	0.60 ***	-

*Note*. *** *p* ≤ 0.001; ** *p* ≤ 0.01; * *p* < 0.05; PC = perfectionistic concerns, SAPS Discrepancy scale; PS = perfectionistic strivings, SAPS Standards scale; SPS = Study-related Perfectionism Scale; PSWQ = Penn State Worry Questionnaire; P-OCS = Parents’ Overstudy Climate Scale; T-OSC = Teachers’ Overstudy Climate Scale; SH = Studyholism; SE = Study Engagement; GPA = grade point average; H-Gen = hours of study per day generally; H-Exams = hours of study per day before exams; Drop Int. = dropout intention; PANAS+ = positive affect; PANAS− = negative affect; DASS-21 = Depression Anxiety Stress Scale-21, general stress; MSQ-SQ = Mini Sleep Questionnaire, sleep quality; MSQ-DS = Mini Sleep Questionnaire, daytime sleepiness; SRCS-Q = Study-Relationships Conflict Scale, Quarrels at School scale; SRCS-C = Study-Relationships Conflict Scale, Family And Friends’ Complaints scale; SRCS-RI = Study-Relationships Conflict Scale, Relationship Impairment scale.

**Table 3:** Standardized path weights, 95% bias-corrected confidence interval, and *R*^2^ for each dependent variable of the path analysis model (*n* = 793).

Dependent Variable	*R* ^2^	Predictor	*β*	*p*	95% Bias-Corrected C.I. with Related *p*	In Line with College Students *
Study-Perfectionism	0.22					
		Perf. Strivings	0.27	<0.001	[0.22; 0.34], 0.003	Yes
		Perf. Concerns	0.14	<0.001	[0.07; 0.22], 0.003	Yes
		Worry	0.25	<0.001	[0.18; 0.33], 0.005	Yes
		**T-OSC**	**0.002**	**ns**	**[−0.06; 0.07], ns**	**No ^a^**
Studyholism	0.48					
		Perf. Strivings	0.04	ns	[−0.02; 0.10], ns	Yes
		Perf. Concerns	0.04	ns	[−0.01; 0.11], ns	Yes
		Study-Perfectionism	0.02	ns	[−0.04; 0.08], ns	Yes
		Worry	0.62	<0.001	[0.56; 0.68], 0.004	Yes
		P-OSC	0.04	ns	[−0.03; 0.09], ns	Yes
		**T-OSC**	**0.10**	**<0.001**	**[0.05; 0.15], 0.005**	**No ^b^**
		Professional School	–0.06	0.04	[−0.11; 0.01], ns	N/A
		Technical School	0.01	ns	[−0.05; 0.06], ns	N/A
Study Engagement	0.29					
		Perf. Strivings	0.41	<0.001	[0.34; 0.46], 0.005	Yes
		Perf. Concerns	–0.24	<0.001	[0.−30; −0.17], 0.005	Yes
		Study-Perfectionism	0.21	<0.001	[0.15; 0.30], 0.002	Yes
		**Worry**	**0.11**	**<0.001**	**[0.04; 0.19], 0.004**	**No ^c^**
		P-OSC	0.03	ns	[−0.03; 0.10], ns	Yes
		T-OSC	–0.01	ns	[−0.07; 0.06], ns	Yes
		Professional School	0.01	ns	[−0.07; 0.07], ns	N/A
		Technical School	–0.02	ns	[−0.08; 0.04], ns	N/A
Grade Point Average	0.26					
		**Perf. Strivings**	**0.17**	**0.004**	**[0.11; 0.25], 0.002**	**No ^d^**
		Perf. Concerns	–0.18	<0.001	[−0.25; −0.09], 0.005	Yes
		Study-Perfectionism	0.10	<0.001	[0.03; 0.18], 0.008	Yes ^e^
		Studyholism	–0.06	ns	[−0.13; 0.01], ns	Yes
		Study Engagement	0.31	<0.001	[0.22; 0.38], 0.007	Yes
		Professional School	–0.20	<0.001	[−0.28; −0.13], 0.004	N/A
		Technical School	–0.11	<0.001	[−0.18; −0.06], 0.002	N/A
Hours of study per day—Generally	0.30					
		Studyholism	0.23	<0.001	[0.15; 0.30], 0.008	Yes ^f^
		Study Engagement	0.34	<0.001	[0.28; 0.41], 0.006	Yes
		Study-Perfectionism	0.01	ns	[−0.06; 0.07], ns	Yes
		Professional School	–0.25	<0.001	[−0.32; −0.19], 0.005	N/A
		Technical School	–0.24	<0.001	[−0.30; −0.16], 0.005	N/A
Hours of study per day—Before Exams	0.22					
		Studyholism	0.29	<0.001	[0.23; 0.36], 0.004	Yes ^g^
		Study Engagement	0.22	<0.001	[0.16; 0.29], 0.004	Yes
		Study-Perfectionism	–0.03	ns	[−0.10; 0.05], ns	Yes
		Professional School	–0.22	<0.001	[−0.29; −0.14], 0.004	N/A
		Technical School	–0.19	<0.001	[−0.26; −0.13], 0.002	N/A
Dropout Intention	0.18					
		Studyholism	0.32	<0.001	[0.23; 0.39], 0.005	Yes
		Study Engagement	–0.30	<0.001	[−0.37; −0.23], 0.002	Yes
		**Perf. Concerns**	**0.05**	**ns**	[−0.03; 0.11], ns	**No ^h^**
		Professional School	0.19	<0.001	[0.12; 0.26], 0.003	N/A
Positive Affect	0.14					
		Studyholism	–0.02	ns	[−0.12; 0.08], ns	Yes
		Study Engagement	0.15	<0.001	[0.06; 0.22], 0.005	Yes
		Perf. Strivings	0.29	<0.001	[0.21; 0.35], 0.005	Yes
		Perf. Concerns	–0.06	ns	[−0.14; 0.01], ns	Yes
		**Worry**	**–0.06**	**ns**	[−0.15; 0.05], ns	**No ^i^**
Negative Affect	0.21					
		Studyholism	0.10	0.02	[0.01; 0.19], 0.036	Yes ^l^
		**Study Engagement**	**–0.14**	**<0.001**	[−0.20; −0.07], 0.005	**No ^m^**
		Perf. Concerns	0.18	<0.001	[0.11; 0.26], 0.004	Yes
		Worry	0.28	<0.001	[0.19; 0.37], 0.003	Yes
General Stress	0.39					
		Studyholism	0.13	0.001	[0.02; 0.19], 0.028	Yes ^n^
		Study Engagement	–0.18	<0.001	[−0.23; −0.11], 0.008	Yes ^o^
		Perf. Concerns	0.14	<0.001	[0.07; 0.20], 0.004	Yes
		Worry	0.47	<0.001	[0.39; 0.54], 0.003	Yes
Sleep Quality Impairment	0.21					
		Studyholism	0.14	0.002	[0.03; 0.24], 0.010	Yes
		**Study Engagement**	**–0.15**	**<0.001**	**[−0.22; −0.08], 0.003**	**No ^p^**
		Perf. Concerns	0.15	<0.001	[0.07; 0.22], 0.005	Yes
		Worry	0.27	<0.001	[0.18; 0.37], 0.002	Yes
Daytime Sleepiness	0.22					
		Studyholism	0.22	<0.001	[0.12; 0.30], 0.007	Yes
		Study Engagement	–0.15	<0.001	[−0.22; −0.09], 0.004	Yes
		Perf. Concerns	0.15	<0.001	[0.06; 0.21], 0.006	Yes
		Worry	0.20	<0.001	[0.11; 0.30], 0.004	Yes
Quarrels at School	0.11					
		Studyholism	0.07	ns	[−0.02; 0.15], ns	Yes
		**Study Engagement**	**–0.20**	**<0.001**	**[−0.28; −0.13], 0.004**	**No ^q^**
		**Study-Perfectionism**	**0.08**	**0.03**	**[0.02; 0.15], 0.019**	**No ^r^**
		T-OSC	0.18	<0.001	[0.11; 0.24], 0.004	Yes ^s^
		Professional School	0.18	<0.001	[0.10; 0.25], 0.005	N/A
Family and Friends’ Complaints	0.19					
		**Studyholism**	**–0.02**	**ns**	**[−0.08; 0.05], ns**	**No ^t^**
		Study Engagement	0.31	<0.001	[0.24; 0.37], 0.004	Yes
		Study-Perfectionism	0.24	<0.001	[0.17; 0.31], 0.003	Yes
Relationship impairment	0.22					
		Studyholism	0.29	<0.001	[0.22; 0.36], 0.005	Yes
		Study Engagement	0.17	<0.001	[0.10; 0.24], 0.003	Yes
		Study-Perfectionism	0.18	<0.001	[0.11; 0.25], 0.004	Yes
		Professional School	–0.01	ns	[−0.06; 0.06], ns	N/A
		Technical School	–0.04	ns	[−0.10; 0.01], ns	N/A

*Note*. * Loscalzo and Giannini’s (2019) study [5]; bold font = there is a difference between the results found in the youths and adolescents samples; C.I. = confidence interval; Perf. = perfectionistic; Study-perfectionism = study-related perfectionism; P-OSC = parents’ overstudying climate; T-OSC = teachers’ overstudying climate; N/A = not applicable; ^a^ = in the college student sample, T-OSC (in the form of overt comments about the performance, not in the form of pressure towards hard study) is a positive predictor; ^b^ = in the college student sample, the *β* value is 0.08 for the pressure towards hard study, which is, however, under the 0.10 selected cut-off value; ^c^ = in the college student sample, the *β* value is 0.07; ^d^ = in the college student sample, the *β* value is 0.08; ^e^ = in the college student sample, the *β* value is higher (0.21); ^f^ = in the college student sample, the *β* value is lower (0.15); ^g^ = in the college student sample, the *β* value is lower (0.14); ^h^ = in the college student sample, it is a positive predictor; ^i^ = in the college student sample, it is a negative predictor; ^l^ = in the college student sample, the *β* value is higher (0.20); ^m^ = in the college student sample, the *β* value is –0.08; ^n^ = in the college student sample, the *β* value is higher (0.25); ^o^ = in the college student sample, the *β* value is lower (–0.11); ^p^ = in the college student sample, the *β* value is –0.08; ^q^ = in the college student sample, the *β* value is –0.08; ^r^ = in the college student sample, the *β* value is 0.13; ^s^ = in the college student sample, the *β* value is 0.27 for the overt comments form; ^t^ = in the college student sample, it is a positive predictor.

**Table 4:** Mann–Whitney tests for Studyholism antecedents and outcomes by Studyholic type.

Dependent Variable	*U*	*Z*	*p*	*r*	Type of Student	*Median*	*n*
Perf. Strivings	48.50	−3.92	<0.001	–0.61	Disengaged Studyholic	11.00	14
					Engaged Studyholic	20.00	27
					Total	15.00	752
Perf. Concerns	181.50	–0.21	ns	–0.03	Disengaged Studyholic	14.00	14
					Engaged Studyholic	15.00	27
					Total	12.00	752
Study-perfectionism	66.00	–0.3.38	0.001	–0.53	Disengaged Studyholic	21.50	14
					Engaged Studyholic	32.00	27
					Total	24.00	752
Worry	176.50	–0.34	ns	–0.05	Disengaged Studyholic	65.50	14
					Engaged Studyholic	64.00	27
					Total	47.00	752
P-OSC	169.50	–0.54	ns	–0.08	Disengaged Studyholic	22.00	14
					Engaged Studyholic	19.00	27
					Total	19.00	752
T-OSC	177.00	–0.33	ns	–0.05	Disengaged Studyholic	22.00	14
					Engaged Studyholic	21.00	27
					Total	19.00	752
Sleep Quality ^#^	133.50	−1.53	ns	–0.24	Disengaged Studyholic	22.00	14
					Engaged Studyholic	17.00	27
					Total	14.50	752
Daytime Sleepiness	75.50	−3.13	0.002	–0.49	Disengaged Studyholic	23.00	14
					Engaged Studyholic	15.00	27
					Total	16.00	752
Positive Affect	151.00	−1.05	ns	–0.16	Disengaged Studyholic	26.00	14
					Engaged Studyholic	29.00	27
					Total	28.00	752
Negative Affect	96.00	−2.56	0.01	–0.40	Disengaged Studyholic	29.50	14
					Engaged Studyholic	18.00	27
					Total	17.00	752
General Stress	123.00	−1.82	ns	–0.28	Disengaged Studyholic	34.50	14
					Engaged Studyholic	30.00	27
					Total	18.00	752
Quarrels at School	97.00	−2.58	0.01	–0.40	Disengaged Studyholic	7.00	14
					Engaged Studyholic	4.00	27
					Total	4.00	752
Fam. Friends Comp.	80.50	−3.08	0.002	–0.48	Disengaged Studyholic	3.00	14
					Engaged Studyholic	6.00	27
					Total	4.00	752
Rel. Impairment	122.50	−1.86	ns	–0.29	Disengaged Studyholic	3.00	14
					Engaged Studyholic	8.00	27
					Total	4.00	752
Hours-Generally	39.50	−4.20	<0.001	–0.66	Disengaged Studyholic	2.00	14
					Engaged Studyholic	3.50	27
					Total	2.00	744
Hours-Exams	129.50	−1.66	ns	0.26	Disengaged Studyholic	4.00	14
					Engaged Studyholic	4.00	27
					Total	3.00	747
GPA	89.50	−2.79	0.005	–0.44	Disengaged Studyholic	6.55	14
					Engaged Studyholic	7.00	27
					Total	7.00	747
Dropout Intention	63.50	−3.49	<0.001	–0.55	Disengaged Studyholic	12.50	14
					Engaged Studyholic	5.00	27
					Total	5.00	747

*Note*. Perf. = perfectionistic; Study-perfectionism = study-related perfectionism; P-OSC = parents’ overstudying climate; T-OSC = teachers’ overstudying climate; ^#^ = a higher score indicates greater sleep quality impairment; Fam. Friends Comp. = family and friends’ complaints; Rel. Impairment = relationship impairment; Hours-Generally = hours of study per day, generally; Hours-Exams = hours of study per day, before exams; GPA = grade point average.

**Table 5:** Differences between Studyholic types. A comparison with Loscalzo and Giannini’s [5] results.

Variable	Loscalzo and Giannini (2019)	Adolescents
Perf. Strivings	>Engaged Studyholic	>Engaged Studyholic
**Perf. Concerns**	**<Engaged Studyholic**	**No difference**
Study-perfectionism	>Engaged Studyholic	>Engaged Studyholic
Worry	No difference	No difference
P-OSC	No difference	No difference
T-OSC	No difference	No difference
Sleep Quality ^#^	No difference	No difference
**Daytime Sleepiness**	**No difference**	**<Engaged Studyholic**
**Positive Affect**	**>Engaged Studyholic**	**No difference**
Negative Affect	<Engaged Studyholic	<Engaged Studyholic
General Stress	No difference	No difference
**Quarrels at School**	**No difference**	**<Engaged Studyholic**
Fam. Friends Comp.	>Engaged Studyholic	>Engaged Studyholic
**Rel. Impairment**	**>Engaged Studyholic**	**No difference**
Hours-Generally	>Engaged Studyholic	>Engaged Studyholic
**Hours-Exams**	**>Engaged Studyholic**	**No difference**
GPA	>Engaged Studyholic	>Engaged Studyholic
Dropout Intention	<Engaged Studyholic	<Engaged Studyholic

*Note*. Bold font = there is a difference between the results found in the youths’ and adolescents’ samples; Perf. = perfectionistic; Study-perfectionism = study-related perfectionism; P-OSC = parents’ overstudy climate; T-OSC = teachers’ overstudy climate; ^#^ = a higher score indicates greater sleep quality impairment; Fam. Friends Comp. = family and friends’ complaints; Rel. Impairment = relationship impairment; Hours-Generally = hours of study per day, generally; Hours-Exams = hours of study per day, before exams; GPA = grade point average; >, Higher; <, Lower.

## Data Availability

The data presented in this study are available on request from the corresponding author.
